# Resistance intensity status of *Anopheles gambiae s.l.* species at KOLOKOPE, eastern plateau Togo: A potential site to assess new vector control tools

**DOI:** 10.1016/j.heliyon.2022.e09770

**Published:** 2022-06-22

**Authors:** Koffi Mensah Ahadji-Dabla, Joseph Chabi, Yawo Georges Apetogbo, Edoh Koffi, Melinda Patricia Hadi, Guillaume Koffivi Ketoh

**Affiliations:** a Laboratory of Ecology and Ecotoxicology, Department of Zoology, Faculty of Sciences, Université de Lomé, 01 B.P: 1515 Lomé 01, Togo; b Department of Parasitology, Noguchi Memorial Institute for Medical Research (NMIMR), University of Ghana, P.O. Box LG 581, Legon, Accra, Ghana; c Vestergaard Frandsen East Africa, Nairobi, Kenya

**Keywords:** Next-generation LLINs, Resistance intensity, *Anopheles gambiae s.l.*, Piperonyl butoxide

## Abstract

According to WHO recommendations, the deployment of the next generation of Long-Lasting Insecticidal Nets (LLINs) for malaria vector control requires appropriate investigations on the insecticide resistance profile of the vector. Most of the next generation of LLINs are impregnated with a combination of pyrethroid insecticides and piperonyl butoxide (PBO), a synergist with an additional impact on the increase in the mortality rate of *Anopheles gambiae s.l.* (Diptera: Culicidae). Kolokopé is a cotton-growing area in the central region of Togo characterized by an intensive use of agricultural pesticides and insecticides where there is a phase II experimental hut station. For the characterization of the site, WHO susceptibility tests using diagnostic doses of ten insecticides, PBO synergist assays and intensity assays of three pyrethroids (5x and 10x) were conducted on adult female mosquitoes obtained from larvae collected around the site. *Anopheles gambiae s.l.* from Kolokopé showed high resistance to pyrethroids and DDT, but to a lesser extent to carbamates and organophosphates. Likewise, high intensity of resistance to pyrethroid was observed with less than 40% mortality at 10x deltamethrin, 52 and 29% mortality at 10x permethrin and 10x alphacypermethrin, respectively. Also, PBO treatment resulted in increased mortality which was higher than the mortality rate at 10x doses of pyrethroids. The high pyrethroid intensity resistance recorded at Kolokopé could be mainly due to the selection pressure on *An. gambiae s.l.* caused by the excessive use of insecticide in agriculture. These results can be used to assess the next generation of LLINs either in experimental hut or at a community trial.

## 1 Introduction

The spread of insecticide resistance in malaria vectors has been a source of constant concerns for malaria endemic countries. In African countries south of the Sahara, insecticide resistance in *Anopheles gambiae s.l.* (mainly *An. gambiae*, *An. coluzzii*) threatens the large distribution of Long-Lasting Insecticidal Nets (LLINs) and Indoor Residual Spraying (IRS) campaigns [1]. The next generation of LLINs are nets manufactured using piperonyl butoxide (PBO) combined with a pyrethroid, incorporated into the fibers during the producing process. The use of pyrethroids including deltamethrin, permethrin, and cypermethrin through different application methods, puts considerable resistance selection pressure on many pests of importance in public health, particularly on malaria vectors [2]. Piperonyl butoxide is a synergist that inhibits specific enzymes including cytochrome P450 which detoxify pyrethroids in mosquitoes [2]. Recently, the World Health Organization (WHO) recommended the Pyrethroid-PBO nets following its interim endorsement as a new vector control tool in 2017, to be deployed by countries in areas where mosquitoes are resistant to pyrethroid [3]. Before their approval by the WHO, LLINs were subjected to an evaluation in experimental huts. Experimental hut trials also known as Phase II trials are designed in accordance with standard outcome measures to assess LLINs efficacy in inhibiting blood-feeding, deterrence, induced exophily, and mortality in mosquitoes. In fact, the use of pyrethroids through different application methods, puts considerable resistance selection pressure on many pests of importance in public health, particularly on malaria vectors [4]. In 2017, 76 malaria endemic countries that reported standard monitoring data for 2010 to 2016, showed that resistance was detected in 61 (54%) countries to at least one insecticide in one malaria vector from a collection site. The same report stated that from 2010 to 2016, malaria endemic countries that reported pyrethroid resistance increased from 71 to 81% [5].

Pyrethroids have been used since the 1980's, and the resistance to this class of insecticide was reported in many countries, especially in West Africa [6, 7, 8, 9, 10, 11], and it had impacted LLINs vector control strategies. In these countries, there is a need to develop and deploy new tools, as stated by the third pillar of the Global Plan for Insecticide Resistance Management in Malaria Vectors (GPIRM), launched in 2012 by the WHO [12]. Malaria vectors developed several mechanisms allowing them to be less susceptible to pyrethroids. These include the *kdr* mutation (*kdr* L1014F in West Africa and *kdr* L1014S in East Africa), the *ace1* mutation (a substitution from glycine to serine at position 119, also called mutation G119S), and increased detoxification enzyme activity. Therefore, in some areas, malaria vector populations subjected to the diagnostic doses show a decreased susceptibility to pyrethroids and to many other insecticides.

There is therefore, an urgent need to develop new agents or combination of insecticides to control resistant mosquitoes. Eight years ago, the efficiency of pyrethroid-PBO nets including PermaNet® 3.0 and Olyset® Plus was evaluated in Phase II experimental huts at Kolokopé [11], and currently, the National Malaria Control Program (NMCP) of Togo is planning to test a new molecule for IRS in the same area. Also, the effectiveness of the new molecules for IRS and tools (LLINs) in controlling resistance in mosquitoes need to be well assessed in appropriate sites and countries using standard procedures such as the Phase II hut trials.

This study was therefore initiated to characterize and profile the *An. gambiae s.l.* mosquitoes from Kolokopé, where about seven standard experimental huts have been set up to evaluate new vector control tools.

## 2 Material and methods

### 2.1 Study site

Kolokopé is a village located in the Plateau region of Togo (07°47′59″N, 01°18′E), about 200 km from Lomé, the capital city. The region is characterized by a long rainy season from March to October and a dry season from November to February. The annual rainfall is estimated at 1300–1500mm per year. Over the year, the average temperature in Kolokopé is around 27 °C. Farming activities, specifically cotton cultivation with approximately 236 hectares of land and an estimated production of 1000 tons per year, are the main source of revenue of the population. Excessive or inappropriate quantity of insecticides is used in the area for crop protection [13], and resistance to deltamethrin and permethrin were recently reported in the area [11].

### 2.2 WHO susceptibility test

Susceptibility testing of *An. gambiae s.l.* populations from Kolokopé was conducted using the WHO test kits according to standard testing protocols [14]. Four classes of insecticides, including (1) pyrethroids (0.05% deltamethrin, 0.75% permethrin, 0.05% lambdacyhalothrin, and 0.05% alphacypermethrin), (2) organochlorine (4% DDT), (3) organophosphates (1% fenitrothion, 5% malathion, and 0.25% pirimiphos methyl), and (4) carbamates (0.1% propoxur and 0.1% bendiocarb) were tested at the diagnostic concentrations (DC). Additionally, synergist assays were performed using the synergist PBO 4% (Sigma-Aldrich, USA) with DC of deltamethrin and permethrin and intensity assays using 5x and 10x diagnostic doses of deltamethrin, permethrin, and alphacypermethrin [15]. These insecticides were selected because they are used to impregnate pyrethroid-PBO nets such as PermaNet® 3.0, Olyset® Plus, and Veeralin® LN, respectively. Regarding intensity assays, 98–100% mortality rates at 5×DC indicates that there is no need for assay at the 10×DC (low resistance intensity); mortality rates less than 98% at the 5×DC (moderate resistance intensity, 10×DC assay needed); 98–100% mortality rates at the 10×DC indicates a moderate resistance intensity; and finally, high resistance intensity is indicated by mortality rates lower than 98% at 10×DC. All impregnated papers were purchased from WHO Press, World Health Organization.

Because the study was not a longitudinal trial and it was performed few days prior to the evaluation of LLIN efficacy in the experimental huts in Kolokopé, only one-time collection was done. *Anopheles gambiae s.l.* larvae collected around the study site in October 2017, were brought to the field insectary and reared in 30 × 15 × 10 cm breeding tanks. Pupae were collected every day in paper cups and put into 30 × 30 × 30 cm cages for adult emergence under standard conditions (25 ± 2 °C, 80 ± 4% relative humidity (RH)). The adult females were then used for the susceptibility test. Four batches of 20–25 non-blood fed females (F_0_) aged 3–5 days were exposed for an hour under ambient conditions (27 ± 2 °C and 75 ± 5% RH) to different doses of above-mentioned insecticides’ impregnated papers. In the synergist test, mosquitoes were first exposed to PBO for an hour before being exposed to the insecticide for an additional 1 h. Before the exposure to impregnated paper, mosquitoes were inserted and kept for an hour into holding tubes, they were then transferred to the exposure tubes for a period of 1 h. The exposure tubes were placed in a reduced lighting area to reduce light intensity. At the end of the 1-hour exposure period, the mosquitoes were transferred back to the holding tubes, provided 10% sugar water contained in a pad of a soaked cotton wool placed on the mesh-screen and kept for 24 h. The number of mosquitoes knocked down was recorded every 10 min during the exposure time, and the mortality rates were recorded after 24 h [16]. Tests with silicone and olive oil impregnated papers serving as controls were run in parallel. The susceptible *An. gambiae*, Kisumu strain was used as reference.

### 2.3 Mosquito collection in experimental huts

During the same period of larvae collection (October 2017), mosquitoes were collected in the experimental huts constructed in the study site for *Anopheles* species-specific PCR analyses. Adult volunteers were recruited among the inhabitants of the villages, given the objectives of the study clearly explained in their local language by an interpreter before they signed the informed consent. Sleeper volunteers slept under the net set up in each hut. Each volunteer entered a hut at dusk and in the morning, and collected mosquitoes from the hut using mouth aspirator. In case any confirmed case of *P. falciparum* parasitaemia was detected, the patient was immediately treated with Coartem (artemether 20mg/lumefantrine 120 mg).

### 2.4 Species identification and kdr L1014F and ace1 G119S detection

*Anopheles* specimens from the susceptibility testing stored at -20 °C and those collected from experimental huts were randomly selected for PCR analyses. SINE-PCR was used for species identification [17]. The detection of *kdr* L1014F and *ace1* G119S alleles was conducted following the methods of Martinez-Torres et al. [18] and Weill et al. [19], respectively.

DNA extraction was done from a whole mosquito using the protocol designed by Collins et al. [18] and amplified in 20μl of a master mixture. The master mixture consisted of Taq Buffer (5X, 4μl), MgCl2 (25 mM, 2 μl), dNTPs (5 mM, 0.8 μl), Primer F (20 μM, 0.3 μl), Primer R (20 μM, 0.3 μl), Taq DNA polymerase (5U/μl, 0.07 μl), ddH20, and DNA extract (3 μl). 5′-TCG-CCT TAG ACC TTG CGT TA-3′ and 5′-CGC TTC AAG AAT TCG AGA TAC-3'are the two specific primers used for this procedure. Regarding the amplification conditions, DNA polymerase activation was performed with an initial step for 10 min at 94 °C followed by 35 cycles with denaturation at 94 °C for 30s. Hybridization was done at 54 °C for 30s and at 72 °C for 1min and finally, elongation was done at 72 °C for 10min followed by a decrease in temperature to 4 °C. Amplified fragments were analyzed by electrophoresis on 2% agarose gels stained with ethidium bromide and visualized under UV light. The weight bands corresponded to fragments containing or lacking the targeted SINE200.

The detection *kdr* L1014F mutation was performed with common primers, Agd1 (5′-ATA GAT TCC CCG ACC ATG-3′) and Agd2 (5′-AGA CAA GGA TGA TGA ACC-3′), susceptible primer, Agd3 (5′-AAT TTG CAT TAC TTA CGA CA-3′), and resistance primer, Agd4 (5′-CTG TAG TGA TAG GAA ATT TA-3′). The composition of the master mixture is as follows: Taq Buffer (5X, 2.5 μl), MgCl2 (25 mM, 0.5 μl), dNTP (10 μM, 0.5 μl), Primer Agd1 (10 μM, 0.3 μl), Primer Agd2 (10 μM, 0.3 μl), Primer Agd3 (10 μM, 1 μl), Primer Agd4 (10 μM, 1 μl), ddH20 (6.35 μl), Taq DNA polymerase (0,25 U/μl, 0.05 μl), and DNA extract (2 μl). DNA polymerase activation (94 °C for 3s followed by 35 cycles at 94 °C for 30s) was followed by the hybridization (30 s at 55 °C and 10 s at 72 °C) and finally the elongation (5 min at 72 °C). The expected band sizes, to distinguish resistant and susceptible in sibling species, are 293 bp fragment for the common band, 195 bp and 137 bp for resistant and susceptible alleles, respectively.

To detect the presence of the *ace1* G119S mutation, a PCR-Restriction Fragment Length Polymorphism (PCR-RFLP) was performed. The master mixture is as follows: 25 μl PCR reaction of 1 μl each of 10 μM Primers EX3AGdir (GATCGTGGACACCGTGTTCG) and EX3AGrev (AGGATGGCCCGCTGGAACAG), 12.5 μl of GoTaq, 9 μl of DNase-free water and 1.5 μl of 1/40 dilution of DNA template. An enzymatic digestion step followed the PCR reaction. A 20 μl restriction enzyme reaction mixture was prepared as follows: 2 μl of Enzymatic Buffer B 10X, 0.2 μl of Acetylated BSA at 10 μg/μl, 0.5 μl of 10 U/μl restriction enzyme Alu I (Promega), 12.3 μl of DNase-free water, and 5 μl of PCR products. Samples were incubated at 37 °C for 4 h in a thermocycler. The resulting products were electrophoresed on 2 % agarose TBE gels stained with ethidium bromide and visualized under UV light to differentiate a 403 bp fragment for susceptible homozygous mosquitoes (SS) and two fragments of 253 bp and 150 bp for homozygous resistant (RR).

### 2.5 Statistical analysis

Mortality rates from the susceptibility bioassay were interpreted according to the WHO criteria:

when mortality rate was ≥98%, individuals were considered susceptible; mortality rate of 90–97% implies suspected resistant; and <90% means confirmed resistant. Two-proportions Z-Test was used in R for comparisons. Mortality rates in DC was compared to 5xDC and 10xDC, respectively and mortality rate in pyrethroid-only was also compared to PBO + pyrethroid. Calculations of the *kdr* L1014F mutation frequency were done using the following formula: F(*kdr*) = 2A + B/2n, with A the number of homozygotes, B the number of heterozygotes, and n the total number of specimens analyzed.

## 3 Results

### 3.1 Resistance status of *Anopheles gambiae s.l*

Using the WHO standard criteria, the wild population of *An. gambiae s.l*. tested is highly resistant to deltamethrin, permethrin, and alphacypermethrin at 10xDC, with mortality rates of 39, 52, and 29%, respectively (Table 1). Significant difference was obtained between DC and 5xDC, and DC and 10xDC, respectively (*p < 0.05*). The mortality rates increase with the exposition of mosquitoes to PBO before the exposition to DCs of deltamethrin and permethrin (*p < 0.05*). Mosquitoes were resistant to the DC of all other insecticides tested except to malathion, where a suspected resistance was observed (96.6%). Also, mosquitoes were susceptible to the DC of pirimiphos methyl. No mortality rate was recorded in the positive controls (silicone and olive oil) and in the Kisumu reference strain.

**Table 1:** Resistance profile of *An. gambiae s.l.* from Kolokopé.

Insecticides	% Mortality						
	Diagnostic concentration (%)	DC (N)	5xDC (N)	10xDC (N)	Status	Silicone/olive oil controls	Kisumu strain
Deltamethrin	0.05	1.0 (96)*a∗*	24.7 (81)*bd*	39.2 (79)*ce*	**Highly Resistant**	0.0 (95)	100.0 (103)
PBO + Deltamethrin	4 + 0.05	98.0 (100)*∗∗*	-	-	Susceptible	0.0 (97)	100.0 (107)
Permethrin	0.75	0.0 (87)a∗	40.8 (76)*bd*	52.1 (73)*cd*	**Highly Resistant**	0.0 (89)	100.0 (99)
PBO + Permethrin	4 + 0.75	62.2 (90)*∗∗*	-	-	Resistant	0.0 (98)	100.0 (97)
Alphacypermethrin	0.05	7.4 (81)a	20.8 (77)*bd*	29.1 (79)*ce*	**Highly Resistant**	0.0 (85)	100.0 (101)
Lambdacyhalothrin	0.05	5.0 (80)	-	-	Resistant	0.0 (90)	100.0 (108)
DDT	4	4.9 (102)	-	-	Resistant	0.0 (87)	100.0 (95)
Bendiocarb	0.1	81.1 (95)	-	-	Resistant	0.0 (92)	100.0 (98)
Propoxur	0.1	80.7 (88)	-	-	Resistant	0.0 (94)	100.0 (96)
Fenitrothion	1	82.8 (93)	-	-	Resistant	0.0 (85)	100.0 (110)
Malathion	5	96.6 (88)	-	-	Suspected Resistant	0.0 (83)	100.0 (102)
Pirimiphos methyl	0.25	100.0 (103)	-	-	Susceptible	0.0 (90)	100.0 (95)

N represents the number of female mosquitoes exposed. In the same line, same letters indicate no significant difference (p > 0.05) and different letters indicate significant difference (p < 0.05). Likewise, asterisks ∗ and ∗∗ in row 3 indicate significant difference (p < 0.05) between pyrethroid-only and PBO + pyrethroid. WHO criteria were applied to all mortality rates.

### *3.2* *Anopheles* species and *kdr* L1014F and *ace1* G119S mutations associated

A total of 176 mosquitoes were successfully identified out of 185 analyzed by PCR. Two species (n = 176) were identified at Kolokopé (*An. gambiae* and *An. coluzzii*) (see Table 2). *Anopheles gambiae* was more frequent both in WHO susceptibility testing and experimental huts samples with 98.8 and 97.9%, respectively. *Anopheles coluzzii* represented less than 1%. The knockdown *L1014F* allele was present at high frequency (>0.9) especially in *An. gambiae*, with 86 individuals over 87 carrying the homozygote RR allele in the WHO susceptibility testing and 83 over 86 in the experimental huts. The *G119S* allele was present at low frequency in both species. In *An. gambiae s.s.*, the *G119S* allele frequency was 0.13 and 0.1 in the WHO susceptibility testing and in the experimental huts, respectively.

**Table 2:** Species composition and resistance mechanisms of *An. gambiae s.l.* from Kolokopé sampled from two different collection methods.

Type of sample	Species	Total	KdrW GENOTYPING					ACE-1 GENOTYPING				
			RR	RS	SS	Total	FREQUENCY	RR	RS	SS	Total	FREQUENCY
WHO susceptibility test	*Anopheles gambiae s.s*	87 (98.8%)	86	0	1	87	0.99	1	19	60	80	0.13
	*Anopheles coluzzii*	1 (0.12%)	1	0	0	1	1.00	0	0	1	1	0.00
	***Total***	**88**	**87**	**0**	**1**	**88**	**0.99**	**1**	**19**	**61**	**82**	**0.13**
Experimental hut	*Anopheles gambiae s.s*	86 (97.7%)	83	1	2	86	0.97	0	17	67	84	0.10
	*Anopheles coluzzii*	2 (0.23%)	0	1	1	2	0.25	0	1	1	2	0.25
	***Total***	**88**	**83**	**2**	**3**	**88**	**0.95**	**0**	**18**	**68**	**86**	**0.10**

## 4 Discussion

According to the Insecticide Resistance Action Committee, resistance is “a heritable change in the sensitivity of a pest population that is reflected in the repeated failure of a product to achieve the expected level of control when used according to the label recommendation for that pest species” [20]. Based on the mortality rates recorded at 10×DC, this study showed that the resistance status of *An. gambiae s.l.* to pyrethroids was far beyond the WHO recommended diagnostic doses. Cotton production is the main agriculture practice at Kolokopé with an estimated production of 1000 tons per year [13]; such activity requires the use of a significant quantity of insecticides and fertilizers. A study by Yadouleton et al. [21] reported a regular use of insecticides in cotton fields in Benin; therefore, insecticide residues are accumulated in the soil during crop treatment and are drawn in the breeding sites water bodies [9, 22]. Resistance is then selected at the mosquito's larval stage. Hien et al. [22] reported the presence of toxic compounds in the water of the conventional cotton site in Burkina Faso. In the same country, high kdr frequency was detected in cotton-growing areas than in only food crops-growing rural areas that are not treated with insecticide [8]. In a recently published study, three kdr mutations (L1014F, L1014S, and N1575Y) were detected at Nangbeto, a neighboring site [23, 24].

Monitoring activities of *An. gambiae s.l.* resistance to insecticide are being conducted in several areas of Togo and showed high level of pyrethroid resistance at Kolokopé [9, 22] or its surrounding areas [25]. This study reports high pyrethroid intensity resistance in *An. gambiae s.l*. at Kolokopé which is similar to recent reports from Ghana [26], Mali [27], and Nigeria [28]. It is attributed to selection pressure on vector populations following the rapid scale-up and use of pyrethroid-based vector control interventions and the use of pyrethroid insecticides in agriculture [29].

To date in Togo, malaria vector control relies exclusively on the use of LLINs, which is one of the strategies recommended by the WHO [30]. Therefore, through national and routine campaigns, the NMCP of Togo had distributed a total of 4,706,417 LLINs in 2017. The important result obtained in this study is the high level of pyrethroid resistance. This situation sounds the bell for an urgent implementation of insecticide resistance management program. The good news, however, is that Kolokopé is now the best area to assess the efficacy of new malaria vector control tools using the experimental huts constructed for that purpose. We can hypothesize that any tool that could efficiently control the *Anopheles* strain of Kolokopé, could as well control at least the other wild strains across the country.

The susceptibility tests clearly showed that when the mosquitoes were exposed to PBO prior to pyrethroids, their mortality rate increased. Study reports showed that PBO is a synergist that inhibits the mixed function oxidases (MFO), P450s, and esterases involve in pyrethroid resistance in mosquitoes [31]. Though we did not perform biochemical assays of detoxifying enzymes in each *Anopheles* population in this study, the intensity resistance recorded using only pyrethroid could be explained by high kdr mutation frequency, MFO activities, and an increase in esterase activity. Experimental hut trials conducted at Kokolopé in 2013 revealed the efficacy of two LLINs: PermaNet® 3.0 and Olyset® Plus, these LLINs are PBO + deltamethrin and PBO + permethrin incorporated, respectively [9]. Also, a study by Dadzie et al. [32] in Ghana, reported the role of PBO in enhancing the efficacy of pyrethroid insecticides against *An. gambiae s.l*.

It is important to emphasize the fact that two species (*An. gambiae* and *An. coluzzii*) were identified in this study with *An. gambiae* being the most represented. However, in a previous study, it was reported that *An. gambiae* and *An. coluzzii* were prevalent at ∼50/50 [11].

## 5 Conclusion

This study reveals the high pyrethroid intensity resistance recorded at Kolokopé which could be mainly due to the pressure on *An. gambiae s.l.* through the excessive use of insecticide in agriculture. Piperonyl butoxide increased pyrethroids mortality rates. This can contribute to the assessment of the next generation of LLINs either in experimental huts or in community trials. Finally, periodic monitoring of the resistance status of malaria vectors in the study area would be useful.

## Declarations

### Author contribution statement

Koffi Mensah Ahadji-Dabla & Joseph Chabi: Conceived and designed the experiments; Performed the experiments; Analyzed and interpreted the data; Wrote the paper.

Yawo ​Georges Apétogbo & Edoh Koffi: Conceived and designed the experiments; Performed the experiments.

Melinda Patricia Hadi: Contributed reagents, materials, analysis tools or data.

Guillaume Koffivi Ketoh: Conceived and designed the experiments; Wrote the paper.

### Funding statement

This research did not receive any specific grant from funding agencies in the public, commercial, or not-for-profit sectors.

### Data availability statement

Data included in article/supplementary material/referenced in article.

### Declaration of interest’s statement

The authors declare no conflict of interest.

### Additional information

No additional information is available for this paper.
